# Association Between Type of Antidiabetic Treatment and Glycemic Control in Patients With Type 2 Diabetes Mellitus: A Retrospective Cross-Sectional Study

**DOI:** 10.7759/cureus.113266

**Published:** 2026-07-23

**Authors:** Rafael Violante-Ortiz, Emanuel Narvaez Gallifa, Erick Eduardo Hernandez Molina, Norma Fernández-Ordóñez, Jose Eugenio Guerra Cardenas, Elizabeth Reyna-Beltrán

**Affiliations:** 1 Endocrinology and Diabetes, Facultad de Medicina de Tampico "Dr. Alberto Romo Caballero", Universidad Autónoma de Tamaulipas, Tampico, MEX; 2 Diabetes Center, Joslin Diabetes Center, Boston, USA; 3 General Medicine, Centro de Estudios de Investigación Metabólicos y Cardiovasculares, S.C., Tampico, MEX; 4 Endocrinology and Metabolism, Centro de Estudios de Investigación Metabólicos y Cardiovasculares, S.C., Tampico, MEX; 5 Obstetrics and Gynecology, Facultad de Medicina de Tampico "Dr. Alberto Romo Caballero", Universidad Autonoma de Tamaulipas, Tampico, MEX; 6 Biological Sciences, Facultad de Medicina de Tampico "Dr. Alberto Romo Caballero", Universidad Autónoma de Tamaulipas, Tampico, MEX

**Keywords:** glycated hemoglobin (hba1c), good glycemic control, ​insulin therapy, oral antidiabetic drugs, type 2 diabetes mellitus (t2dm)

## Abstract

Background: Maintaining adequate glycemic control remains a major challenge in patients with type 2 diabetes mellitus (T2DM). Patients requiring insulin therapy often have longer disease duration and more advanced metabolic deterioration, which may influence the relationship between type of antidiabetic treatment and glycemic control.

Objective: This study aimed to evaluate the association between type of antidiabetic treatment and glycemic control in patients with T2DM.

Methods: This retrospective cross-sectional study analyzed a clinical database of 995 patients with T2DM treated at a specialized metabolic and cardiovascular center in southern Tamaulipas, Mexico. Patients were classified into two treatment groups: oral antidiabetic therapy and combined insulin plus oral antidiabetic therapy. Glycemic control was defined as glycated hemoglobin (HbA1c) ≤7%. Descriptive analyses were performed, and continuous and categorical variables were compared using Student's t-test and chi-squared test, respectively. Multivariable logistic regression was used to evaluate the independent association between type of antidiabetic treatment and glycemic control, adjusting for age, sex, diabetes duration, and body mass index (BMI).

Results: A total of 995 patients were included: 780 (78.4%) received oral antidiabetic therapy, and 215 (21.6%) received combined insulin plus oral antidiabetic therapy. Overall, 192 patients (19.3%) achieved glycemic control, while 803 (80.7%) remained uncontrolled. Glycemic control was achieved in 172 patients (22.1%) in the oral antidiabetic therapy group and 20 patients (9.3%) in the combined insulin plus oral antidiabetic therapy group. Type of antidiabetic treatment was significantly associated with glycemic control (χ²=17.59; p<0.001). In the adjusted logistic regression model, oral antidiabetic therapy was independently associated with higher odds of achieving glycemic control compared with combined insulin plus oral antidiabetic therapy (OR=2.35; 95% CI: 1.41-3.93; p=0.001). Longer diabetes duration was negatively associated with glycemic control, whereas BMI was not independently associated.

Conclusions: Type of antidiabetic treatment was significantly associated with glycemic control in patients with T2DM. However, the lower probability of glycemic control among patients receiving combined insulin plus oral antidiabetic therapy likely reflects longer disease duration and greater disease severity rather than reduced treatment effectiveness. These findings highlight the importance of early optimization of diabetes management before progression to advanced disease requiring insulin therapy.

## Introduction

Type 2 diabetes mellitus (T2DM) is a major global health problem and one of the leading causes of chronic morbidity worldwide. The International Diabetes Federation estimated that 589 million adults were living with diabetes in 2024, with projections suggesting a continued increase over the coming decades [1]. In Mexico, diabetes represents a particularly important public health challenge, with national survey data reporting a high prevalence of both diagnosed and undiagnosed diabetes among adults [2].

Maintaining adequate glycemic control is central to reducing the risk of diabetes-related complications. Long-term hyperglycemia has been strongly associated with microvascular and macrovascular outcomes in patients with T2DM [3]. Follow-up data from landmark diabetes studies have also shown that early intensive glucose control may provide durable benefits on diabetes-related outcomes over time [4]. Glycated hemoglobin (HbA1c) remains one of the most widely used markers for long-term glycemic assessment, and an HbA1c goal below 7% is commonly recommended for many nonpregnant adults, although individualized targets are emphasized according to age, comorbidities, hypoglycemia risk, and treatment burden [5].

Pharmacologic management of T2DM typically begins with lifestyle modification and oral antidiabetic therapy, with treatment intensification considered when glycemic targets are not achieved. Current consensus recommendations emphasize a patient-centered approach that considers cardiovascular and renal comorbidities, hypoglycemia risk, weight, cost, access, and patient preferences when selecting glucose-lowering therapy [6]. As beta-cell function progressively declines, many patients eventually require injectable therapy, including insulin, frequently in combination with oral antidiabetic agents [6,7].

However, patients receiving combined insulin plus oral antidiabetic therapy often represent a clinically different population. They frequently have longer diabetes duration, greater metabolic deterioration, higher treatment complexity, and more advanced disease. Therapeutic inertia and delays in treatment intensification may further contribute to prolonged exposure to hyperglycemia before insulin is initiated [8]. Therefore, worse glycemic control among insulin-treated patients in observational studies may reflect disease severity, treatment delay, or selection bias rather than lower treatment effectiveness.

Previous studies have identified several factors associated with poor glycemic control, including longer diabetes duration, medication adherence, body mass index (BMI), treatment complexity, and use of insulin or combined therapy [9,10]. In addition, therapeutic inertia has been described as an important barrier to timely treatment intensification in patients with T2DM [11,12]. Nevertheless, the relationship between treatment modality and glycemic control remains clinically relevant, particularly in real-world settings where treatment groups are not randomized and may differ substantially in baseline characteristics.

Understanding how treatment modality is associated with glycemic control in routine clinical practice may help identify patients at higher risk of inadequate control and support earlier therapeutic optimization. Therefore, this study aimed to evaluate the association between treatment modality and glycemic control in patients with T2DM treated at a specialized metabolic and cardiovascular center, using a retrospective cross-sectional analysis adjusted for clinically relevant variables.

## Materials and methods

Study design and setting

This retrospective cross-sectional study was conducted through a secondary analysis of a clinical database from CAD Diabetes Care Center, a specialized metabolic and cardiovascular center in Tampico, Tamaulipas, Mexico. Data were collected from clinical records dated between May 20, 2024, and April 30, 2026. The database included patients with T2DM who received outpatient care at the center. The study was designed to evaluate the association between type of antidiabetic treatment and glycemic control in routine clinical practice.

Study population

A total of 1,509 clinical records with patient identifiers were initially assessed for eligibility. Patients were included if they had a documented diagnosis of T2DM, available HbA1c data, available clinical and anthropometric information, and a clearly documented type of antidiabetic treatment. Only patients treated with oral antidiabetic therapy alone or combined insulin plus oral antidiabetic therapy were included. Records were excluded if they had missing or incomplete data for the main outcome variable, unclear treatment classification, a diagnosis other than T2DM, duplicate or nonvalid entries, or treatment regimens outside the two study groups. After applying the inclusion and exclusion criteria, 995 patients were included in the final analysis. 

Variables and definitions

The primary outcome was glycemic control, which was determined using HbA1c as the main outcome indicator. Patients with HbA1c ≤7% were classified as having adequate glycemic control, whereas those with HbA1c >7% were classified as uncontrolled. The main independent variable was type of antidiabetic treatment. The oral therapy group included patients treated with oral antidiabetic agents only, while the combined therapy group included patients treated with insulin plus oral antidiabetic agents.

Additional covariates included age, sex, duration of T2DM, systolic blood pressure, diastolic blood pressure, height, weight, BMI, and BMI category. BMI was calculated as weight in kilograms divided by height in meters squared. For analysis, BMI was categorized using three predefined adult BMI groups, namely, BMI <25 kg/m², 25-29.9 kg/m², and ≥30 kg/m², corresponding to normal or underweight, overweight, and obesity, respectively.

Statistical analysis

Data were analyzed using IBM SPSS Statistics for Windows, Version 27.0 (IBM Corp., Armonk, New York, United States). Continuous variables were assessed for normality using the Shapiro-Wilk test and visual inspection of histograms and Q-Q plots. Homogeneity of variances between groups was assessed using Levene's test. Continuous variables were summarized as means and standard deviations, while categorical variables were presented as frequencies and percentages. Because of the large sample size, continuous variables were compared between treatment groups using Student's t-test, with consideration of variance homogeneity. Categorical variables were compared using Pearson's chi-squared test.

The association between type of antidiabetic treatment and glycemic control was first evaluated using Pearson's chi-squared test. Cramer's V was calculated to estimate the strength of the association. A multivariable logistic regression model was then performed to evaluate the independent association between type of antidiabetic treatment and glycemic control. The dependent variable was glycemic control, coded as 0 for uncontrolled and 1 for controlled. Independent variables included in the model were type of antidiabetic treatment, age, sex, duration of T2DM, and BMI. Results were reported as odds ratios with 95% confidence intervals. A p-value of <0.05 was considered statistically significant.

## Results

A total of 1,509 clinical records were assessed for eligibility. After applying the inclusion and exclusion criteria, 514 records were excluded, and 995 patients with T2DM were included in the final analysis. Of these, 780 (78.4%) were treated with oral antidiabetic therapy alone, while 215 (21.6%) received combined insulin plus oral antidiabetic therapy. Baseline demographic and clinical characteristics according to type of antidiabetic treatment are shown in Table 1.

**Table 1:** Baseline demographic and clinical characteristics according to type of antidiabetic treatment Values are presented as mean±SD or n (%). Continuous variables were compared using Student's t-test, and categorical variables were compared using Pearson's chi-squared test. The test statistic column reports t-values (t) for continuous variables and chi-squared values (χ²) for categorical variables. BMI categories were defined as follows: normal, BMI <25 kg/m²; overweight, BMI 25-29.9 kg/m²; and obesity, BMI ≥30 kg/m². BMI: body mass index; HbA1c: glycated hemoglobin; SD: standard deviation; T2DM: type 2 diabetes mellitus

Characteristic	Oral antidiabetic therapy (n=780)	Combined insulin plus oral antidiabetic therapy (n=215)	Test statistic	P-value
Age (years), mean±SD	54.43±12.08	58.28±11.54	t=-4.18	<0.001
Duration of T2DM (years), mean±SD	8.42±8.23	16.06±9.15	t=-11.07	<0.001
Systolic blood pressure (mmHg), mean±SD	135.59±19.44	134.70±21.61	t=0.55	0.586
Diastolic blood pressure (mmHg), mean±SD	81.78±10.65	79.11±11.36	t=3.20	0.001
Height (m), mean±SD	1.60±0.10	1.58±0.10	t=2.77	0.006
Weight (kg), mean±SD	77.36±18.10	72.35±16.96	t=3.64	<0.001
BMI (kg/m²), mean±SD	29.96±5.81	28.87±6.00	t=2.41	0.016
HbA1c (%), mean±SD	9.15±2.32	9.94±2.19	t=-4.46	<0.001
Sex, n (%)				
Male	296 (37.9%)	57 (26.5%)	χ²=9.63	0.002
Female	484 (62.1%)	158 (73.5%)		
BMI category, n (%)				
Normal weight	150 (19.2%)	50 (23.3%)	χ²=5.04	0.080
Overweight	285 (36.5%)	88 (40.9%)		
Obesity	345 (44.2%)	77 (35.8%)		

Patients receiving combined insulin plus oral antidiabetic therapy were older than those receiving oral therapy alone and had a longer duration of T2DM. The combined therapy group also had higher HbA1c levels and a lower mean BMI. Sex distribution differed significantly between groups, with a higher proportion of women in the combined insulin plus oral antidiabetic therapy group. BMI category distribution did not differ significantly between treatment groups.

Overall, 192 patients (19.3%) achieved adequate glycemic control, whereas 803 (80.7%) were uncontrolled. Glycemic control according to type of antidiabetic treatment is shown in Table 2.

**Table 2:** Association between type of antidiabetic treatment and glycemic control Glycemic control was defined as HbA1c ≤7%. Percentages are presented by treatment group. Pearson's chi-squared test showed a statistically significant association between type of antidiabetic treatment and glycemic control (χ²=17.59; p<0.001). Cramer's V was 0.133. HbA1c: glycated hemoglobin

Type of antidiabetic treatment	Uncontrolled n (%)	Controlled n (%)	Total
Oral antidiabetic therapy	608 (77.9%)	172 (22.1%)	780
Combined insulin plus oral antidiabetic therapy	195 (90.7%)	20 (9.3%)	215
Total	803 (80.7%)	192 (19.3%)	995

The proportion of patients achieving glycemic control was higher in the oral antidiabetic therapy group than in the combined insulin plus oral antidiabetic therapy group. Although the difference was statistically significant, the strength of association was small based on Cramer's V.

A multivariable logistic regression model was performed to evaluate the independent association between type of antidiabetic treatment and glycemic control after adjustment for age, sex, duration of T2DM, and BMI. The results are shown in Table 3.

**Table 3:** Multivariable logistic regression for glycemic control The dependent variable was glycemic control, coded as 0 for uncontrolled and 1 for controlled. The model was adjusted for type of antidiabetic treatment, age, sex, duration of T2DM, and BMI. The reference group for type of antidiabetic treatment was combined insulin plus oral antidiabetic therapy, and the reference group for sex was female. OR: odds ratio; CI: confidence interval; BMI: body mass index; T2DM: type 2 diabetes mellitus

Variable	Adjusted OR	95% CI	P-value
Oral antidiabetic therapy vs. combined insulin plus oral antidiabetic therapy	2.35	1.40-3.93	0.001
Age	1.028	1.013-1.044	<0.001
Sex, male vs. female	1.00	0.72-1.41	0.982
Duration of T2DM	0.966	0.944-0.988	0.003
BMI	1.017	0.989-1.046	0.226

In the adjusted model, oral antidiabetic therapy was independently associated with higher odds of achieving glycemic control compared with combined insulin plus oral antidiabetic therapy. Age was positively associated with glycemic control, while longer duration of T2DM was negatively associated. Sex and BMI were not independently associated with glycemic control.

## Discussion

The main finding of this study was that type of antidiabetic treatment was significantly associated with glycemic control in patients with T2DM. Patients receiving oral antidiabetic therapy had a higher proportion of adequate glycemic control compared with those receiving combined insulin plus oral antidiabetic therapy. This association remained statistically significant after adjustment for age, sex, duration of T2DM, and BMI. However, these findings should not be interpreted as evidence that oral antidiabetic therapy is superior to combined insulin plus oral antidiabetic therapy. Rather, they likely reflect important clinical differences between treatment groups, particularly longer diabetes duration and more advanced disease among patients requiring insulin.

Patients receiving combined insulin plus oral antidiabetic therapy had a substantially longer duration of T2DM than those treated with oral antidiabetic therapy alone. This finding is clinically relevant because T2DM is a progressive disease in which maintaining glycemic targets becomes increasingly difficult over time. Current recommendations emphasize individualized, patient-centered treatment selection and timely intensification when glycemic goals are not achieved [5,6]. Therefore, patients requiring insulin therapy frequently represent a clinically more complex population with more advanced metabolic deterioration, higher treatment burden, and greater difficulty achieving glycemic control.

In the present study, the lower proportion of glycemic control observed among patients receiving combined insulin plus oral antidiabetic therapy should be interpreted in relation to the clinical characteristics identified in the analysis. This group had a longer duration of T2DM and higher HbA1c levels at baseline, suggesting a more advanced stage of disease and greater difficulty achieving glycemic targets. In the multivariable model, duration of T2DM remained independently associated with lower odds of glycemic control, supporting its relevance as one of the main indicators related to inadequate metabolic control in this sample. These findings are consistent with recent evidence showing that longer diabetes duration and greater treatment complexity are associated with poorer glycemic outcomes in patients with T2DM [13-17].

The association between longer duration of T2DM and poorer glycemic control was also observed in the adjusted regression model. Each additional year of diabetes duration was associated with lower odds of adequate glycemic control. This finding is consistent with recent evidence identifying longer diabetes duration as an important factor associated with poor glycemic control [15-17]. These findings reinforce the importance of early optimization of glucose-lowering therapy before patients progress to more advanced stages requiring insulin.

Age was positively associated with glycemic control in the adjusted model. Although this association was statistically significant, it should be interpreted cautiously. Older patients may have closer medical follow-up, greater adherence to treatment, or more frequent clinical monitoring. However, this study did not evaluate adherence, frequency of visits, socioeconomic factors, comorbidities, or treatment intensity. Therefore, the association between age and glycemic control may be influenced by unmeasured factors. Individualized glycemic targets are recommended in clinical practice, particularly when considering age, comorbidities, hypoglycemia risk, and treatment burden [5].

BMI was not independently associated with glycemic control after adjustment. Although BMI differed between treatment groups in the baseline analysis, its effect was no longer significant in the multivariable model. This suggests that, in this sample, diabetes duration and type of antidiabetic treatment were more strongly associated with glycemic control than BMI. Poor glycemic control in T2DM is multifactorial and may be influenced by treatment complexity, medication adherence, comorbidities, diabetes duration, healthcare access, and follow-up patterns [15-17].

An important clinical implication of these findings is that patients receiving combined insulin plus oral antidiabetic therapy should be recognized as a high-risk group for inadequate glycemic control. These patients may benefit from closer follow-up, structured diabetes education, assessment of adherence, optimization of insulin regimens, and identification of barriers to treatment. Clinical inertia has also been reported as a relevant barrier to treatment intensification in patients with T2DM, supporting the need for earlier recognition of patients at risk of persistent inadequate glycemic control [18]. In real-world clinical practice, identifying patients with longer diabetes duration, higher HbA1c levels, and greater treatment complexity may help guide closer follow-up and individualized therapeutic optimization.

This study has several limitations. First, its retrospective cross-sectional design prevents establishing causal relationships between type of antidiabetic treatment and glycemic control. Second, the study was conducted at a single specialized metabolic and cardiovascular center, which may limit the generalizability of the findings to other clinical settings. Third, the analysis did not include potentially relevant variables such as medication adherence, insulin dose, type of insulin regimen, specific oral antidiabetic agents, renal function, comorbidities, socioeconomic status, diet, physical activity, or frequency of follow-up. Fourth, glycemic control was assessed using HbA1c at a single point in time, and longitudinal changes in glycemic control could not be evaluated. Finally, residual confounding is possible despite multivariable adjustment.

Despite these limitations, this study provides real-world evidence on the association between type of antidiabetic treatment and glycemic control in a large sample of patients with T2DM. The findings emphasize that patients receiving combined insulin plus oral antidiabetic therapy often represent a clinically more complex group with longer disease duration and a lower probability of meeting glycemic targets. Therefore, treatment comparisons in observational studies should be interpreted in the context of disease severity, treatment complexity, and baseline clinical differences.

## Conclusions

In this retrospective cross-sectional study of patients with T2DM, type of antidiabetic treatment was significantly associated with glycemic control. Patients receiving oral antidiabetic therapy had higher odds of achieving adequate glycemic control compared with those receiving combined insulin plus oral antidiabetic therapy, even after adjustment for age, duration of T2DM, and BMI. However, this finding should not be interpreted as evidence that oral antidiabetic therapy is superior to combined insulin plus oral antidiabetic therapy, since patients requiring insulin had a longer duration of diabetes and likely represented a more clinically advanced population.

Longer duration of T2DM was independently associated with lower odds of glycemic control, emphasizing the progressive nature of the disease and the importance of timely therapeutic optimization. These findings highlight the need for early treatment intensification, individualized follow-up, and closer monitoring of patients requiring combined insulin plus oral antidiabetic therapy in order to improve glycemic outcomes in real-world clinical practice.
